# Leaving no one behind: Pakistan’s risk communication and community engagement during COVID-19

**DOI:** 10.7189/jogh.11.03091

**Published:** 2021-07-31

**Authors:** Zaeem ul Haq, Zafar Mirza, Tajudeen Oyeyemi Oyewale, Faisal Sultan

**Affiliations:** 1 Ministry of National Health Services, Regulations & Coordination, Islamabad, Pakistan; 2 United Nations Children’s Fund (UNICEF), Islamabad, Pakistan

Pakistan’s successful flattening of the successive COVID-19 curves is being acknowledged, along with questions being asked for unpacking the factors that contributed to its success [1]. With a fragmented health system, when COVID-19 happened, Pakistan had to scramble its response to the imminent challenge. The need to communicate was urgent, as promotion of behaviours like physical distancing and wearing of face masks is integral to curtailing the virus transmission. Against this backdrop, Pakistan set up the risk communication and community engagement (RCCE) and implemented multi-pronged strategic interventions. The RCCE has been a strong pillar of Pakistan’s response to COVID-19, and one of the contributors to its success. This short report provides a summary of lessons learned during the past year.

## LOCAL SETTING

Pakistan is a low to middle-income country (LMIC), having a population of 220 million spread across four provinces and two regions. After the devolution of health to provinces, the Ministry of National Health Services, Regulations, and Coordination (NHSR&C) has specific roles including, but not limited to, developing a vision for the health sector, interprovincial coordination, and meeting international obligations, eg, International Health Regulations (IHR).

Geographically sandwiched between China and Iran, two countries that faced the initial onslaught of SARS-CoV-2, Pakistan had high vulnerability yet low preparedness. The World Health Organization’s joint appraisal in 2016 assessed that the country was not fully prepared to prevent, detect and respond to health threats [2]. Like other components, RCCE was also assessed as unsatisfactory; ready-to-implement strategies, trained human resources, and adequate funds were scant. The country’s communication landscape is a mixed bag - about 53% of Pakistanis watch television, 16% read newspapers, and 6% listen to the radio [3]. Mobile phone and internet access, however, are vastly available with about 85% and 46% Pakistanis respectively using these services.

## APPROACH

In the early phase of the response, the Ministry of NHSR&C constituted an RCCE Taskforce comprised of members from health and other ministries, line departments, and development partners. The Taskforce was supported by the Inter-Services Public Relations (ISPR); the media wing of the Pakistan Armed Forces. In late March 2020, the National Command and Operations Centre (NCOC) was formed to broaden the spectrum of coordination and response between federal and provincial levels. A data-driven, coherent, and targeted response was made possible.

**Figure 1:**
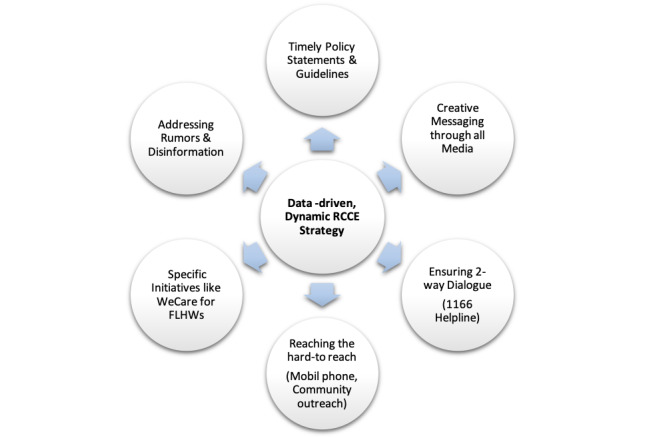
Six streams of the risk communication and community engagement (RCCE) strategic framework implemented in Pakistan.

The collective effort led to a dynamic RCCE approach (**Figure 1**) characterized by regular examination of the COVID-19 data and behavioural insights for strategic response adjustment. The evolving epidemiological, as well as psycho-behavioural picture, informed the communication decisions guided by the social constructionist approach, whereby risk is seen to be interrelated with sociocultural context and not just a scientific measure [4]. Global guidance, adapted to the local context was implemented while maximizing the opportunities offered by different communication platforms.

The strategy guided the following six interconnected streams.

### 1. Policy statements to inform the public in real-time

Keeping people informed about an emergency, and Government’s relevant actions build trust [5]. Pressers every day or on alternate days, that were broadcast on national networks, addressed the evolving nature of the pandemic in real-time. High-level officials informed the public about the epidemiological situation, relevant policy decisions, and the actions that were required on the part of the citizens. Guidelines (SOPs) that addressed emerging situations, were summarized in the presser, and their English and Urdu versions were posted on the websites.

### 2. Public service messages

Creative concepts to promote priority health behaviors ie, mask-wearing, handwashing, and maintaining physical distance were presented through animations and live-action videos, for broadcast on electronic and social media. Testimonials of health workers, religious scholars, athletes, and celebrities were also disseminated. Theoretical guidance from behaviour change models like Social Norms Theory, or Health Belief Model was used to develop these persuasive messages [6].

**Figure:**
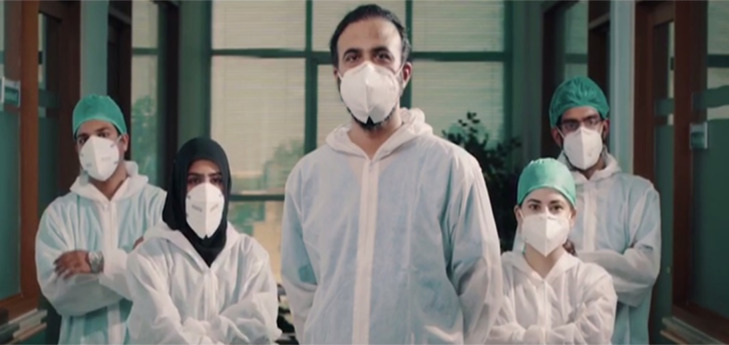
Photo: *We Care* aimed at improving the clinical capacity of Frontline Health Workers while also keeping their morale high through motivational messages (screengrab from a TV advertisement, with permission from Ministry of NHSR&C).

### 3. Ensuring two-way dialogue

While giving out quick updates was important, a 2-way dialogue was crucial for responding to questions, educating the people and building trust. Trust in government communication in turn, is associated with a higher likelihood of people adopting preventive behaviours [5]. To ensure trust through dialogue, Pakistan started a toll-free, call-in helpline during the first month of the pandemic, which went on to receive over 25 000 calls per day during the next year and continues till today.

### 4. Reaching the hard to reach

Reaching everyone is crucial as a lack of information results in several disparities in a pandemic setting [5]. Since relatively few Pakistanis have access to TV and social media, the focus was on the 183 million mobile phone users in an effort to reach as many people as possible. This was achieved through recorded voice messages, used as call-waiting ringtones. The carefully developed messages advised the dos and don’ts in the evolving context. Rural communities that may not have even the mobile phone were reached through vaccinators via mobile miking from their motorbikes, or mosques before the call for prayer (adhan), five times a day.

### 5. Caring for frontline health workers

Using targeted interventions to address pockets of populations that may have unique context and information needs, is critical [5]. With an increase in COVID-19 cases and hospitalizations, an increasing number of Frontline Health Workers (FLHWs) became infected. Moreover, worry of disease and death started inducing aggressive behaviours among the community towards the FLHWs. Responding to this, a campaign *We Care* was launched, comprised of skill-based training of all cadre of health workers on the rational use of Personal Protective Equipment (PPE) in their setting, attending to their need for psychological support, and building a positive social environment of mutual care between medics and community.

### 6. Rumour management

Rumours are unavoidable in a pandemic [7]. Monitoring all forms of media enabled us to spot misinformation and fake news, and respond accordingly. Care was practiced that rumours are not just countered, but a deeper look is taken and the underlying issue addressed where possible [8]. An institutional mechanism was also established along with the Ministry of Interior to deal with the accounts that consistently generated fake information that could harm the people.

## RELEVANT CHANGES

In a country that did not have a formal risk communication mechanism, a vibrant RCCE platform is now available (Table 1). Led by the Health Ministry and the NCOC, several ministries, their departments and programs, development partners, the private sector, and media organizations are important contributors to this platform. The mechanism is geared to continue addressing the current pandemic as well as future emergencies. In today's era of digital communication, partnerships with Facebook, Google, and WhatsApp have matured for long-term, implementation strategies.

**Table 1:** Changes observed relevant to the streams of risk communication and community engagement (RCCE) interventions

Intervention	Dose & reach	Outcome/impact	
Fortnightly RCCE briefs for data-driven, dynamic strategy	36 RCCE briefs for key decision-makers	Evidence-informed RCCE decisions, including some system-level decisions.	
Policy statements & Guidelines (SOPs), respectively broadcast and uploaded to websites	Daily or alternate-day pressers reaching 130 million users. Set of 64 guidelines (SOPs) uploaded in English & Urdu	Govt. actions trusted by 50% Pakistanis	
Public service messages (PSM) based on several theoretical constructs	50 PSMs for 130 million TV viewers multiple times/d, also uploaded to social media	>12 000 min of free TV airtime, >43 million views on social media, 85% of 43 million Facebook users accessed NHSRC pages.
Mobile phone ringtones (call-waiting voice message) and text messages	12 ringtones reaching 167 million mobile phone users every day, and 16 text messages with a circulation of 1.7 billion.	On average, 35% believed others were practicing physical distance; 57% believed others were adhering to face masks.
Community outreach through health workers, mosque, and community elders	Over 8000 vaccinators and health workers and 300 000 community leaders engaged to reach a population of 35 million	60%-80% reported compliance to physical distance, face mask, and handwashing.
Toll free, call-in helpline	250 call agents and 20 doctors responded to a daily average of 30 000 calls (13 million calls in one year)	Questions about rumours, quarantine, home isolation, testing, treatment were addressed. Key insights from calls also informed the overall RCCE strategy.	
Specific initiatives (*We Care* for FLHW)	100 000 FLHWs trained on the appropriate use of PPEs	Average daily new infections among FLHWs reduced during 2 peaks 166 (June 2020) to 36 (May 2021)	
	4 videos for training and morale boosting		
Rumour management through dynamic monitoring and response system	Daily sentiment analysis and debunking, along with content removal, when required	Disinformation around dead body burial, plasma therapy, irrational use of COVID-19 medicines, and misplaced beliefs on traditional remedies addressed.	

RCCE – risk communication & community engagement, FLHW – frontline health worker, PPE – personal protective equipment

Consistent and coherent messages that informed people in real-time, have built and maintained a high level of trust in the Government’s response decisions. The 1166 helpline-originally for polio programme- is institutionalized, becoming a permanent interface between the people and the public health care system.

Outcomes-wise, several waves of independent surveys from credible institutions like Johns Hopkins University documented that about 95% of respondents remembered at least one specific symptom, about 50% of Pakistanis consistently showed trust in Government actions, and between 60%-80% practiced social distancing, face mask, and handwashing regularly [9]. The *We Care* campaign was effective; FLHWs adopted the rational use of PPE because of which the daily new infections among them reduced during the two comparable peaks – 166 per day in June 2020 to 36 per day in May 2021 [10].

## LESSONS LEARNED

Principles from global guidance [7] like coordinated communication, message redundancy, continuous flow of information, tailored messaging, and reaching everyone were implemented, and they worked. Locally, we learned that consistent policy messages coupled with creative messaging helped to remind the population of desired actions. A two-way dialogue is a necessity for people to seek advice and also report their complaints. For low-literate or those with poor access to conventional media, mobile phone-based audio messaging is effective. Information needs of groups having special context must be considered separately, and rumour management is crucial although not easy.

We experienced situations that could become challenging when a centrally designed communication was not possible, eg, spontaneous questions in a provincial or district-level presser, where the capacity of an individual (health worker to a health expert to minister) may not be such that they can readily apply the principles of crisis communication. For example, not being able to simplify technical information for the lay audience, or debunk a rumour was observed. This underscores the need for capacity building at all levels during the preparedness phase.

Lastly, the available RCCE guidance from global sources is insufficient and does not address the several phases of an enormous pandemic like COVID-19, that keep rebounding. Comments have already been made by experts about RCCE reinforcing panic mode rather than diffusing fear [11]. Experience sharing and knowledge management are required to refine the guidance to make it more nuanced, and helpful for countries and programmes in addressing the strategic, tactical, and content development aspects, along with their monitoring and evaluation.
